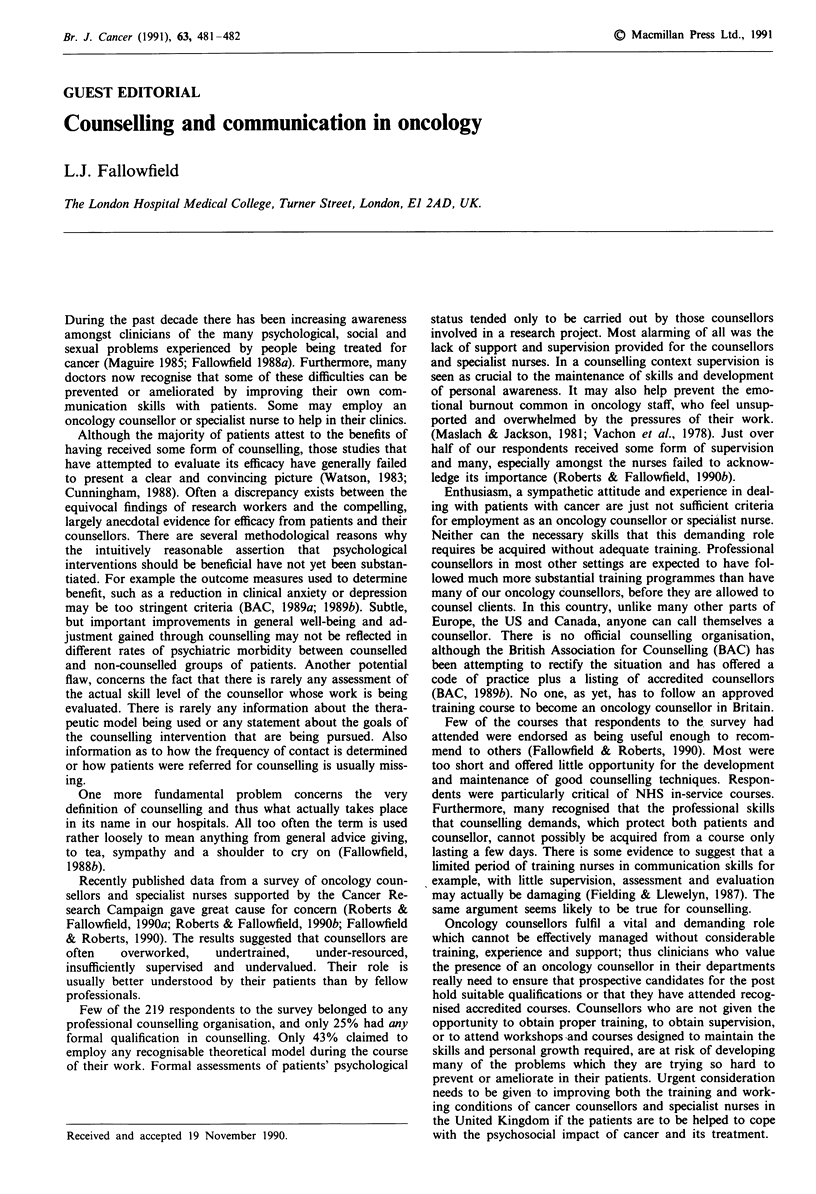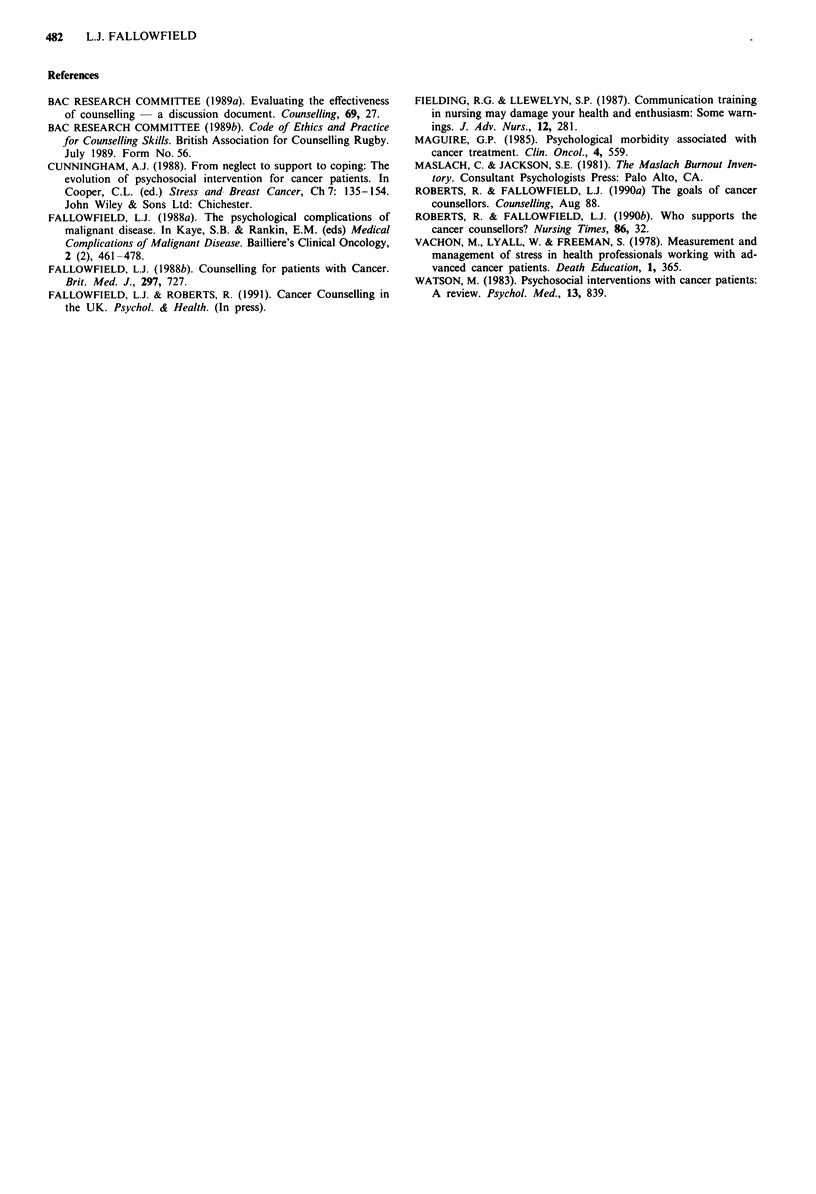# Counselling and communication in oncology.

**DOI:** 10.1038/bjc.1991.114

**Published:** 1991-04

**Authors:** L. J. Fallowfield


Br. J. Cancer (1991), 63, 481–482 © Macmillan Press Ltd., 1991

### GUEST EDITORIAL

### Counselling and communication in oncology

L.J. Fallowfield

*The London Hospital Medical College, Turner Street, London, E1 2AD, UK.*

During the past decade there has been increasing awareness amongst clinicians of the many psychological, social and sexual problems experienced by people being treated for cancer (Maguire 1985; Fallowfield 1988*a*). Furthermore, many doctors now recognise that some of these difficulties can be prevented or ameliorated by improving their own communication skills with patients. Some may employ an oncology counsellor or specialist nurse to help in their clinics.

Although the majority of patients attest to the benefits of having received some form of counselling, those studies that have attempted to evaluate its efficacy have generally failed to present a clear and convincing picture (Watson, 1983; Cunningham, 1988). Often a discrepancy exists between the equivocal findings of research workers and the compelling, largely anecdotal evidence for efficacy from patients and their counsellors. There are several methodological reasons why the intuitively reasonable assertion that psychological interventions should be beneficial have not yet been substantiated. For example the outcome measures used to determine benefit, such as a reduction in clinical anxiety or depression may be too stringent criteria (BAC, 1989*a*; 1989*b*). Subtle, but important improvements in general well-being and adjustment gained through counselling may not be reflected in different rates of psychiatric morbidity between counselled and non-counselled groups of patients. Another potential flaw, concerns the fact that there is rarely any assessment of the actual skill level of the counsellor whose work is being evaluated. There is rarely any information about the therapeutic model being used or any statement about the goals of the counselling intervention that are being pursued. Also information as to how the frequency of contact is determined or how patients were referred for counselling is usually missing.

One more fundamental problem concerns the very definition of counselling and thus what actually takes place in its name in our hospitals. All too often the term is used rather loosely to mean anything from general advice giving, to tea, sympathy and a shoulder to cry on (Fallowfield, 1988*b*).

Recently published data from a survey of oncology counsellors and specialist nurses supported by the Cancer Research Campaign gave great cause for concern (Roberts & Fallowfield, 1990*a*; Roberts & Fallowfield, 1990*b*; Fallowfield & Roberts, 1990). The results suggested that counsellors are often overworked, undertrained, under-resourced, insufficiently supervised and undervalued. Their role is usually better understood by their patients than by fellow professionals.

Few of the 219 respondents to the survey belonged to any professional counselling organisation, and only 25% had *any* formal qualification in counselling. Only 43% claimed to employ any recognisable theoretical model during the course of their work. Formal assessments of patients' psychological status tended only to be carried out by those counsellors involved in a research project. Most alarming of all was the lack of support and supervision provided for the counsellors and specialist nurses. In a counselling context supervision is seen as crucial to the maintenance of skills and development of personal awareness. It may also help prevent the emotional burnout common in oncology staff, who feel unsupported and overwhelmed by the pressures of their work. (Maslach & Jackson, 1981; Vachon *et al*., 1978). Just over half of our respondents received some form of supervision and many, especially amongst the nurses failed to acknowledge its importance (Roberts & Fallowfield, 1990*b*).

Enthusiasm, a sympathetic attitude and experience in dealing with patients with cancer are just not sufficient criteria for employment as an oncology counsellor or specialist nurse. Neither can the necessary skills that this demanding role requires be acquired without adequate training. Professional counsellors in most other settings are expected to have followed much more substantial training programmes than have many of our oncology counsellors, before they are allowed to counsel clients. In this country, unlike many other parts of Europe, the US and Canada, anyone can call themselves a counsellor. There is no official counselling organisation, although the British Association for Counselling (BAC) has been attempting to rectify the situation and has offered a code of practice plus a listing of accredited counsellors (BAC, 1989*b*). No one, as yet, has to follow an approved training course to become an oncology counsellor in Britain.

Few of the courses that respondents to the survey had attended were endorsed as being useful enough to recommend to others (Fallowfield & Roberts, 1990). Most were too short and offered little opportunity for the development and maintenance of good counselling techniques. Respondents were particularly critical of NHS in-service courses. Furthermore, many recognised that the professional skills that counselling demands, which protect both patients and counsellor, cannot possibly be acquired from a course only lasting a few days. There is some evidence to suggest that a limited period of training nurses in communication skills for example, with little supervision, assessment and evaluation may actually be damaging (Fielding & Llewelyn, 1987). The same argument seems likely to be true for counselling.

Oncology counsellors fulfil a vital and demanding role which cannot be effectively managed without considerable training, experience and support; thus clinicians who value the presence of an oncology counsellor in their departments really need to ensure that prospective candidates for the post hold suitable qualifications or that they have attended recognised accredited courses. Counsellors who are not given the opportunity to obtain proper training, to obtain supervision, or to attend workshops and courses designed to maintain the skills and personal growth required, are at risk of developing many of the problems which they are trying so hard to prevent or ameliorate in their patients. Urgent consideration needs to be given to improving both the training and working conditions of cancer counsellors and specialist nurses in the United Kingdom if the patients are to be helped to cope with the psychosocial impact of cancer and its treatment.

Received and accepted 19 November 1990.

### References

BAC RESEARCH COMMITTEE (1989*a*). Evaluating the effectiveness of counselling — a discussion document. *Counselling*, **69**, 27.

BAC RESEARCH COMMITTEE (1989*b*). *Code of Ethics and Practice for Counselling Skills*. British Association for Counselling Rugby. July 1989. Form No. 56.

CUNNINGHAM, A.J. (1988). From neglect to support to coping: The evolution of psychosocial intervention for cancer patients. In Cooper, C.L. (ed.) *Stress and Breast Cancer*, Ch 7: 135-154. John Wiley & Sons Ltd: Chichester.

FALLOWFIELD, L.J. (1988*a*). The psychological complications of malignant disease. In Kaye, S.B. & Rankin, E.M. (eds) *Medical Complications of Malignant Disease*. Bailliere's Clinical Oncology, **2** (2), 461-478.

FALLOWFIELD, L.J. (1988*b*). Counselling for patients with Cancer. *Brit. Med. J.*, **297**, 727.

FALLOWFIELD, L.J. & ROBERTS, R. (1991). Cancer Counselling in the UK. *Psychol. & Health*. (In press).

FIELDING, R.G. & LLEWELYN, S.P. (1987). Communication training in nursing may damage your health and enthusiasm: Some warnings. *J. Adv. Nurs.*, **12**, 281.

MAGUIRE, G.P. (1985). Psychological morbidity associated with cancer treatment. *Clin. Oncol.*, **4**, 559.

MASLACH, C. & JACKSON, S.E. (1981). *The Maslach Burnout Inventory*. Consultant Psychologists Press: Palo Alto, CA.

ROBERTS, R. & FALLOWFIELD, L.J. (1990*a*) The goals of cancer counsellors. *Counselling*, Aug 88.

ROBERTS, R. & FALLOWFIELD, L.J. (1990*b*). Who supports the cancer counsellors? *Nursing Times*, **86**, 32.

VACHON, M., LYALL, W. & FREEMAN, S. (1978). Measurement and management of stress in health professionals working with advanced cancer patients. *Death Education*, **1**, 365.

WATSON, M. (1983). Psychosocial interventions with cancer patients: A review. *Psychol. Med.*, **13**, 839.